# Long-Term Clinical Outcome of Internal Globus Pallidus Deep Brain Stimulation for Dystonia

**DOI:** 10.1371/journal.pone.0146644

**Published:** 2016-01-08

**Authors:** Hye Ran Park, Jae Meen Lee, Gwanhee Ehm, Hui-Jun Yang, In Ho Song, Yong Hoon Lim, Mi-Ryoung Kim, Keyoung Ran Kim, Woong-Woo Lee, Young Eun Kim, Jae Ha Hwang, Chae Won Shin, Hyeyoung Park, Jin Wook Kim, Han-Joon Kim, Cheolyoung Kim, Dong Gyu Kim, Beom Seok Jeon, Sun Ha Paek

**Affiliations:** 1 Department of Neurosurgery, Seoul National University Hospital, Seoul, Republic of Korea; 2 Department of Neurology, Myongji Hospital, Gyeonggi, Republic of Korea; 3 Department of Neurology, Ulsan University Hospital, Ulsan, Republic of Korea; 4 Medical Device Development Center, Osong Medical Innovation Foundation, Chungcheong, Republic of Korea; 5 Department of Neurology, Eulji General Hospital, Seoul, Republic of Korea; 6 Department of Neurology, Hallym University Sacred Heart Hospital, Gyeonggi, Republic of Korea; 7 Department of Neurosurgery, Daejeon Woori Hospital, Gyeonggi, Republic of Korea; 8 Department of Neurology, Seoul National University Hospital, Seoul, Republic of Korea; 9 Medical Imaging Laboratory, and CyberMed, Inc., Seoul, Republic of Korea; 10 Department of Neurosurgery, Seoul National University College of Medicine, Seoul, Republic of Korea; 11 Department of Neurology, Seoul National University College of Medicine, Seoul, Republic of Korea; University of Pennsylvania Perelman School of Medicine, UNITED STATES

## Abstract

**Background:**

GPi (Internal globus pallidus) DBS (deep brain stimulation) is recognized as a safe, reliable, reversible and adjustable treatment in patients with medically refractory dystonia.

**Objectives:**

This report describes the long-term clinical outcome of 36 patients implanted with GPi DBS at the Neurosurgery Department of Seoul National University Hospital.

**Methods:**

Nine patients with a known genetic cause, 12 patients with acquired dystonia, and 15 patients with isolated dystonia without a known genetic cause were included. When categorized by phenomenology, 29 patients had generalized, 5 patients had segmental, and 2 patients had multifocal dystonia. Patients were assessed preoperatively and at defined follow-up examinations postoperatively, using the Burke-Fahn-Marsden dystonia rating scale (BFMDRS) for movement and functional disability assessment. The mean follow-up duration was 47 months (range, 12–84)

**Results:**

The mean movement scores significantly decreased from 44.88 points preoperatively to 26.45 points at 60-month follow up (N = 19, *P* = 0.006). The mean disability score was also decreased over time, from 11.54 points preoperatively to 8.26 points at 60-month follow up, despite no statistical significance (N = 19, *P* = 0.073). When analyzed the movement and disability improvement rates at 12-month follow up point, no significant difference was noted according to etiology, disease duration, age at surgery, age of onset, and phenomenology. However, the patients with DYT-1 dystonia and isolated dystonia without a known genetic cause showed marked improvement.

**Conclusions:**

GPi DBS is a safe and efficient therapeutic method for treatment of dystonia patients to improve both movement and disability. However, this study has some limitations caused by the retrospective design with small sample size in a single-center.

## Introduction

In 2013, an international panel of experts defined dystonia as sustained or intermittent muscle contractions usually causing twisting and repetitive movement or abnormal posture [1–3]. It is one of the most prevalent forms of movement disorder [3]. Dystonia can be classified according to the involved body distribution: focal, segmental, multifocal, generalized, and hemidystonia, or according to the etiology: inherited dystonia of proven genetic origin, acquired dystonia with a known specific cause (e.g., perinatal brain injury, infection, drugs, toxicity, vascular, neoplastic, or brain injury), and isolated dystonia without a known specific cause.

Dystonia may cause considerable morbidity in terms of low self-confidence, pain, depression, and poor social interaction. It has been reported to have a substantial adverse impact on quality of life [4]. Although oral medications and botulinum toxin injections have been the mainstays of treatment for some time, they are not sufficiently effective in some patients.

Remarkable improvement after pallidotomy or pallidal DBS in Parkinson disease (PD) patients suggested a possible benefit of lesioning the GPi as a treatment for dystonia [5]. Trials of bilateral pallidal DBS confirmed this benefit and verified that the procedure can be conducted safely on both sides in one operative session, with promising results in patients with medically refractory dystonia [6, 7]. In recent years, GPi DBS has been employed as a safe, reliable, reversible, and adjustable treatment with a relatively low risk of adverse effects in patients with isolated and acquired dystonia, especially with DYT-1 dystonia [8–16].

However, few studies have investigated the long-term outcome and safety of the GPi DBS. This report describes the long-term clinical outcome of 36 patients implanted with GPi DBS at the Department of Neurosurgery of Seoul National University Hospital.

## Patients & Methods

### Patient population

This study was approved by the institutional review board of Seoul National University Hospital (IRB No. 1505-074-672). The requirement of obtaining written informed consent was waived in consideration of the retrospective study design. From September 2005 until November 2014, a total of 40 patients with medical refractory dystonia underwent DBS surgery at the Department of Neurosurgery of Seoul National University Hospital. One patient who underwent bilateral subthalamic nucleus (STN) DBS and another patient whose surgery had failed due to an intracranial hemorrhage during lead insertion were excluded from this study. Of the remaining patients, 36 patients who received more than 12 months of follow-up were enrolled in this retrospective study. One patient who had a history of failed surgery due to intracranial hemorrhage and received DBS implantation 1 year later was included.

The patients included 26 men and 10 women, with a mean age at surgery of 33 years (range, 10–65). Thirty-three patients underwent bilateral GPi DBS and 3 patients underwent unilateral GPi DBS (right side in 2 cases, left side in 1 case). The mean disease duration before surgery was 91 months (range, 5–380), and the patients were divided into three groups according to the disease duration: less than 36 months, 36–120 months, and more than 120 months. Nineteen patients were adults over 17 years of age at the time of dystonia onset, and 17 patients were children under 16 years of age. When classified by etiology, 9 patients had a known genetic cause of dystonia [3]. Among them, 4 patients had *DYT*-1 gene mutation and 5 patients had pantothenate kinase-associated neurodegeneration (PKAN) with PANK2 gene mutation. Among the 12 patients who had acquired type dystonia, 4 patients had undergone a perinatal brain injury, 4 patients had dystonic cerebral palsy, and 4 patients had drug-induced dystonia (anti-schizophrenic drugs) and were diagnosed as tardive dystonia. The remaining 15 patients were classified as isolated dystonia without a known genetic cause. When categorized by phenomenology, 29 patients had generalized dystonia, 5 patients had segmental dystonia, and 2 patients had multifocal dystonia.

The patients were assessed preoperatively and at defined follow-up examinations postoperatively, at 3, 6, 12, 24, 36, 60, and 84 months. The mean follow-up period was 47 months (range, 12–84). We included the Burke-Fahn-Marsden dystonia rating scale (BFMDRS) for motor and disability assessment. The motor score of the BFMDRS consists of a scale for severity of movement impairment of the eyes, head, speech, trunk, and extremities, with a maximal score of 120 representing the most affected movement [17]. The motor assessment was performed by a specialized neurologist, in an unblinded manner. All patients included in this study gave written informed consent for the surgery and the follow-up examinations and all assessments were performed by neurologists. The written informed consent for the surgery was obtained from the caretakers including parents or next of kin of the minors/children enrolled in this study.

### Surgical procedure

The posteroventral portion of the GPi was targeted by means of axial, sagittal, and coronal MRI images [18]. The theoretical pallidal target was 2 to 4 mm anterior to the midcommissural point, 19 to 22 mm lateral to the midline, and 3 to 6 mm below the intercommissural line. A set of four microelectrodes (Differential microTargeting® Electrodes, FHC, Chemnitz, Germany;1.5 MΩ impedance) were sequentially inserted toward the anatomical target within the GPi, which was chosen vertically on the axial slice at the level of anterior commissure and horizontally at the junction between the two posterior quarters of the GPi [19]. Permanent DBS electrode (DBS 3387, Medtronic, Minneapolis, MN) placement was determined to avoid damage to adjacent vessels, ventricles, and sulci. All procedures were performed under general anesthesia, with the assistance of microelectrode recording (MER). In all cases, the electrode of the left side was inserted earlier than the electrode of the right side, to minimize the error of the dominant side caused by brain shift after cerebrospinal leakage. In consideration of the brain shift, more intraoperative adjustments based on MER were made during the right side electrode positioning. A pulse generator (IPG; Soletra 7426, Medtronic, Minneapolis, MN) was then implanted, and one day after surgery, stimulation parameters were progressively adjusted by telemetry, using an N'vision programmer (Medtronic, Minneapolis, MN). The median values of initial DBS parameters were as follows: pulse width 60 msec (range, 60–180), frequency 130 Hz (range, 60–185), and amplitude 2.5 V (range, 0.5–4.5). S1 Table represents the mean stimulation parameters of the subgroups: DYT-1, PKAN, acquired dystonia, and isolated dystonia without a known genetic cause. We performed a repeat CT scan and fused it with the preoperative MRI to confirm the location of the leads. The analysis about the correlation of the lead location and clinical outcome is in progress.

### Statistical analysis

Statistical analyses were performed using the SPSS 22.0 software (SPSS Inc., Chicago, IL, USA). The mean preoperative and postoperative absolute scores of the BFMDRS and the rate of improvement ([preoperative score—postoperative score]/preoperative score x 100) were calculated. Preoperative and follow-up BFMDRS and functional disability scores were treated as repeated measures for the Wilcoxon signed rank test. The Mann-Whitney-*U* test for unmatched samples was used to compare the percentage of improvement between the preoperative and postoperative conditions. A statistical threshold of *P* < 0.05 (two-tailed) was considered to be significant.

## Results

Table 1 shows the BFMDRS motor and disability scores in the entire patient group that underwent GPi DBS for dystonia, obtained at preoperative baseline and during the follow-up periods. The mean motor scores in the entire patient group significantly decreased overtime (*P* = 0.006), from 44.88 ± 28.12 points preoperatively (N = 36) to 26.45 ± 20.21 points at the 60-month follow up (N = 19). The mean disability score decreased from 11.54 ± 8.16 points preoperatively (N = 36) to 8.26 ± 8.25 points at the 60-month follow up (N = 19); however, the overall reduction in the disability scores did not show statistical significance (*P* = 0.073).

**Table 1:** Overall BFMDRS motor and disability scores of all patients.

	N (cases)	Movement score	Improvement rates (%)	*P*-value^a^
**Preoperative**	36	44.88 ± 28.12		
**3 months**	36	34.12 ± 26.95	28.64 ± 27.14	.000
**6 months**	33	32.02 ± 28.17	36.78 ± 29.25	.118
**12 months**	36	32.76 ± 29.17	32.70 ± 32.18	.000
**24 months**	36	32.40 ± 29.62	38.36 ± 31.69	.000
**36 months**	27	27.67 ± 22.86	34.27 ± 30.32	.000
**60 months**	19	26.45 ± 20.21	31.53 ± 30.63	.033
**84 months**	6	35.75 ± 9.26	29.49 ± 37.61	.345
		*P* = 0.006		
		**Disability score**	**Improvement rates (%)**	***P*-value^a^**
**Preoperative**	36	11.54 ± 8.16		
**3 months**	36	10.17 ± 8.61	22.75 ± 30.35	.138
**6 months**	36	9.64 ± 8.60	27.63 ± 32.31	.069
**12 months**	36	9.93 ± 8.21	24.63 ± 32.03	.095
**24 months**	29	8.24 ± 7.56	27.97 ± 33.33	.075
**36 months**	27	8.67 ± 7.95	27.23 ± 32.60	.520
**60 months**	19	8.26 ± 8.25	27.41 ± 32.73	1.000
**84 months**	7	4.71 ± 4.39	53.92 ± 35.88	.091
		*P* = 0.073		

^a^ This P-value represents the comparison compared to the preoperative value.

The improvement rates of BFMDRS motor and disability scores at the 12-month follow up point were analyzed to determine the impact of etiology, age at surgery, age of onset, disease duration, and phenomenology, as shown in Table 2. The reason for selecting the value at the 12-month follow up for comparison between subgroups was as follows: the maximum beneficial effect in dystonia is known to occur over several weeks after DBS. The patients who were included in this study also obtained benefit since 6 months after DBS. The trend of improvement was steady or slight from 12 months after DBS. The mean motor improvement rate was higher in patients with isolated dystonia without a known genetic cause and inherited dystonia than in those with acquired dystonia (isolated dystonia, 36.71 ± 34.30%; inherited dystonia, 31.33 ± 31.97%; acquired dystonia, 28.73 ± 31.83%; *P* = 0.632). The patients with isolated and inherited dystonia also showed higher disability improvement rates (isolated dystonia, 26.99 ± 34.55%; inherited dystonia, 26.64 ± 34.53%; acquired dystonia, 20.18 ± 29.07%; *P* = 0.238). However, there was no significant difference in the motor and disability improvement rates among the 3 subgroups. Four patients with *DYT*-1 dystonia showed substantially favorable outcomes: motor improvement rate of ~64% and disability improvement rate of ~47%. There was no significant impact of age of onset, age at surgery, disease duration, and phenomenology on movement and disability improvement rates.

**Table 2:** Improvement rates at the 12-month follow-up according to patient characteristics.

Patients group	Movement improvement rates (%)	*P*-value	Disability improvement rates (%)	*P*-value
Etiology		0.632		0.238
Inherited (9 cases)	31.33 ± 31.97		26.64 ± 34.53	
*DYT*-1 (+) (4 cases)	63.76 ± 12.06	0.215	46.67 ± 45.22	0.392
*PKAN* (5 cases)	5.38 ± 6.47	0.846	10.62 ± 11.35	0.177
Acquired (12 cases)	28.73 ± 31.83		20.18 ± 29.07	
Cerebral palsy (4 cases)	37.60 ± 43.42		44.38 ± 51.25	
Perinatal injury (4 cases)	42.91 ± 35.02		19.38 ± 29.61	
Tardive (4 cases)	12.98 ± 25.97	0.994	4.17 ± 4.81	0.601
Idiopathic (15 cases)	36.71 ± 34.30		26.99 ± 34.55	
Age of onset		0.822		0.655
Adult (19 cases, 52.8%)	31.67 ± 31.93		22.51 ± 32.08	
Children (17 cases, 47.2%)	33.86 ± 33.40		27.00 ± 32.78	
Age at surgery		0.822		0.373
Adult (28 cases, 77.8%)	33.51 ± 30.93		26.03 ± 31.85	
Children (8 cases, 22.2%)	29.87 ± 38.42		19.73 ± 34.35	
Disease duration		0.217		0.418
< 36 months (13 cases, 36.1%)	27.10 ± 33.33		20.52 ± 25.22	
36–120 months (14 cases, 38.9%)	32.67 ± 34.84		26.46 ± 40.04	
≥ 120 months (9 cases, 25.0%)	40.85 ± 27.66		27.73 ± 29.74	
Phenomenology		0.905		0.923
Generalized (29 cases, 80.6%)	32.36 ± 32.60		26.79 ± 33.65	
Segmental (5 cases, 13.9%)	34.76 ± 32.94		21.94 ± 26.48	
Multifocal (2 cases, 5.6%)	32.50 ± 45.96			

**Fig 1:**
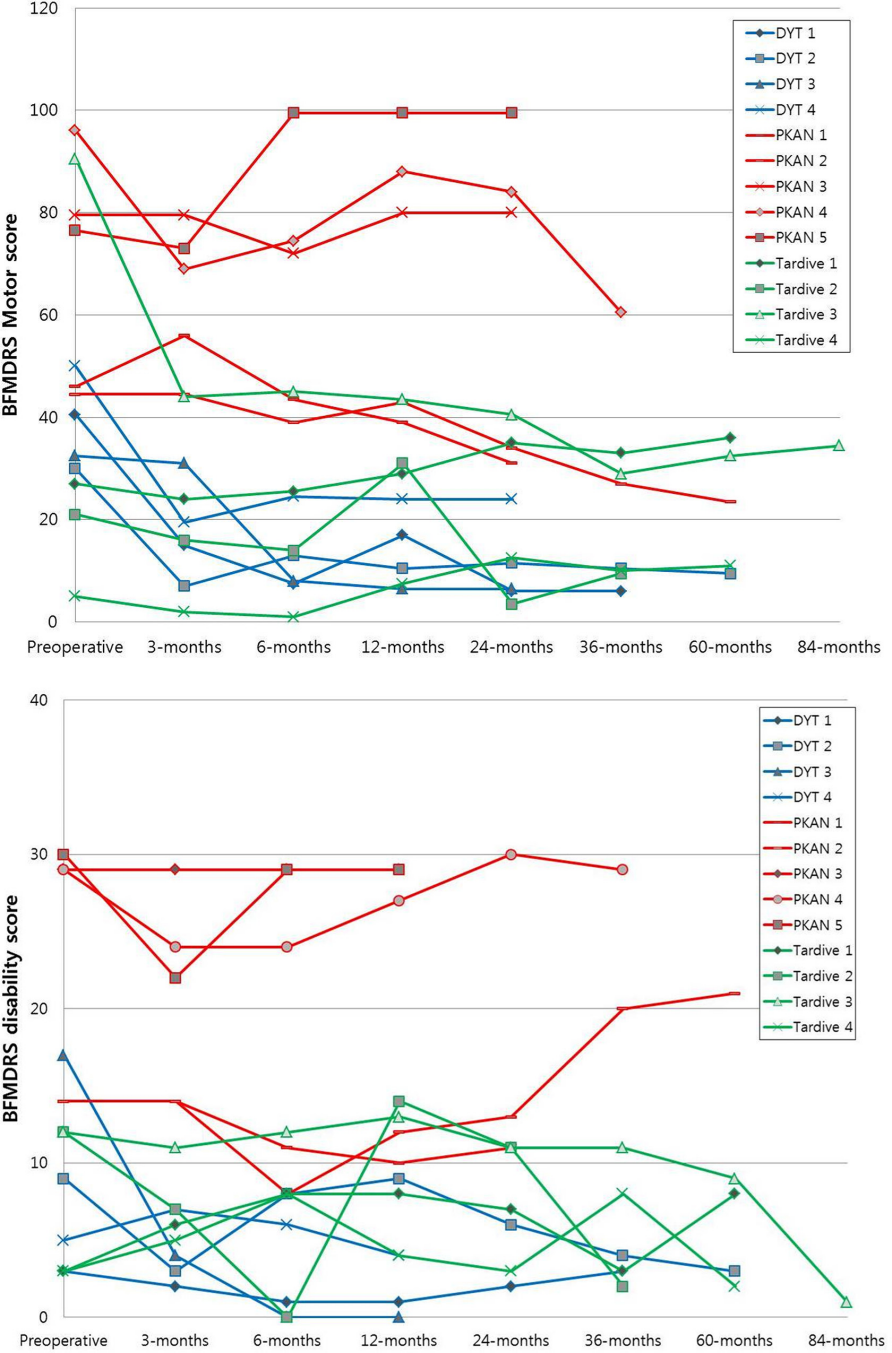
Overall movement and disability scales in the patients with *DYT*-1 dystonia, PKAN, and tardive dystonia. Patients with *DYT*-1 dystonia showed an abrupt decrease in motor and disability scores, and a sustained improved state during the follow-up period. Patients with *PKAN* had relatively higher motor and disability scores preoperatively. But some patients showed substantial improvement in motor score over time; 2 patients acquired improvement which appeared even after postoperative 12 months without resetting (indicated as asterisks). Tardive dystonia patients experienced no considerable improvement in motor and disability scores, except for 1 patient. (PKAN: pantothenate kinase-associated neurodegeneration)

Fig 1 outlines the overall motor and disability scores of the subgroups over time: *DYT*-1 dystonia, PKAN, and tardive dystonia. Patients with *DYT*-1 dystonia showed an abrupt decrease in motor and disability scores, and a sustained improved state during the follow-up period. Patients with *PKAN* had relatively higher motor and disability scores preoperatively. But some patients showed substantial improvement in motor score over time; 2 patients acquired improvement which appeared even after postoperative 12 months without resetting (indicated as asterisks in Fig 1). The stimulation parameters were not changed in these patients. Tardive dystonia patients experienced no considerable improvement in motor and disability scores, except for 1 patient.

## Discussion

### Overall outcome

This report is one of the rare studies on GPi DBS for dystonia with long-term follow up. Previous authors reported that GPi DBS is an effective treatment for dystonia in terms of both movement and disability scores. The results of this study are in close agreement with those obtained by previous authors. Different forms of dystonia are known to show different outcomes, implying that there are differences in the underlying pathophysiology. Previous reports have shown promising results of GPi DBS especially in the patients diagnosed as having PGD with *DYT-*1 positive, focal, and tardive dystonia [9–14, 20, 21]. In this study, patients with isolated dystonia with or without a known genetic cause showed better outcomes than those with acquired dystonia, although the difference was not statistically significant.

Isolated dystonia without a known genetic cause and *DYT*-1 dystonia, which showed favorable outcome after GPi DBS, were classified into primary generalized dystonia (PGD) with or without *DYT*-1. PGD is the only form in which the efficacy of GPi DBS was confirmed in previous studies. In particular, the patients with *DYT*-1 positive PGD were considered to be the group that gains the most benefit from GPi DBS [9–12, 20], although the correlation between the *DYT*-1 gene and clinical outcome is still controversial [22]. Some patients with *DYT*-1 positive PGD showed less improvement than expected [23, 24], and some patients with DYT-1 negative PGD revealed favorable clinical outcomes [14, 25–28]. In the same vein, Jahanshahi et al. recently reported that patients with PGD showed a significant improvement in motor scores regardless of their *DYT*-1 status [29]. Recently, the term “primary” has been replaced by “isolated” [3]. In this study, the mean 12-month motor and disability improvement rates were higher in the *DYT*-1 dystonia group than in those with isolated dystonia without a known genetic cause (motor improvement rate, 63.34 ± 14.74 vs 35.69 ± 35.29, *P* = 0.163; disability improvement rate, 62.22 ± 40.18 vs 22.51 ± 34.79, *P* = 0.046).

**Fig 2:**
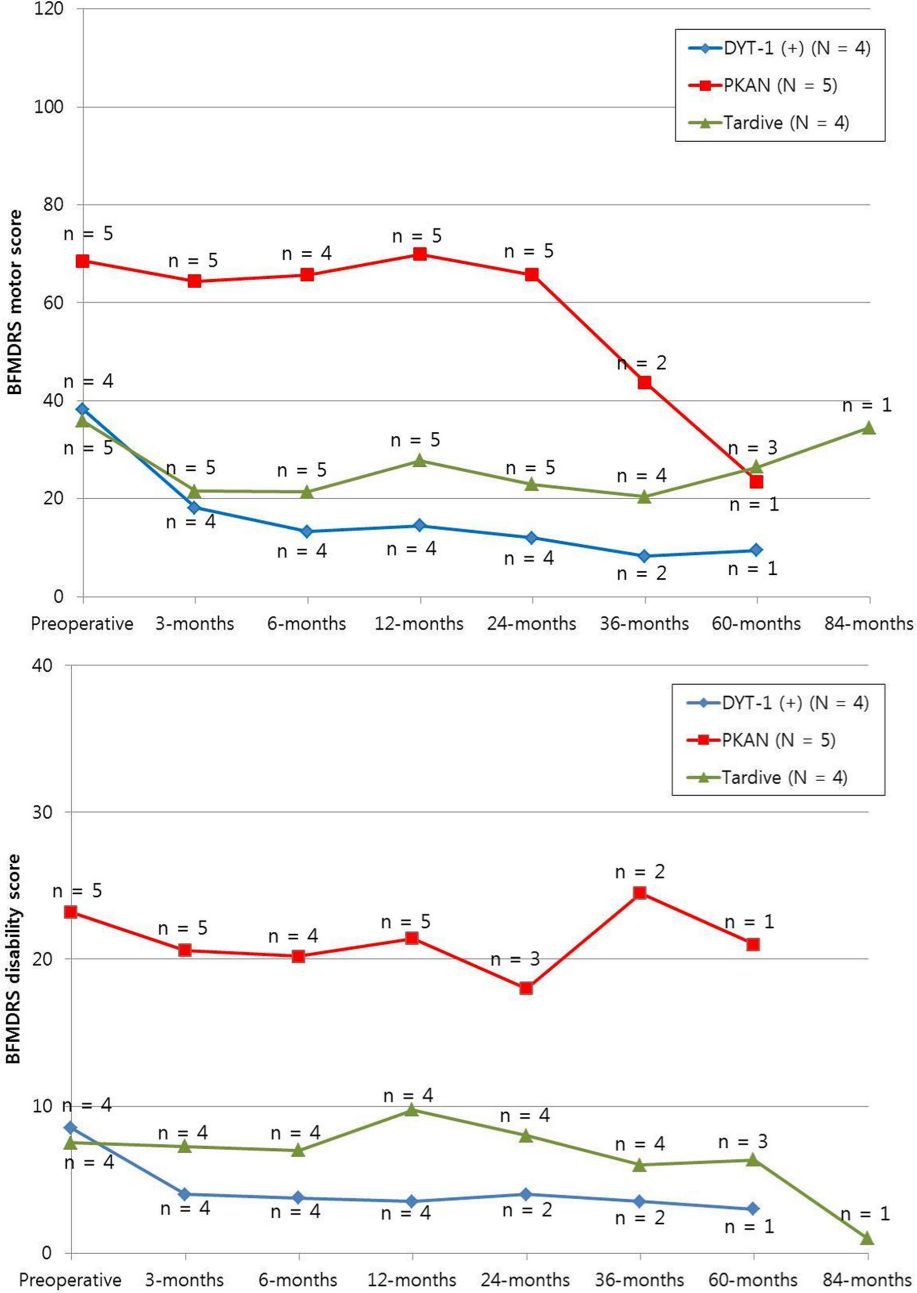
The mean movement and disability scales in the patients with *DYT*-1 dystonia, PKAN, and tardive dystonia. Patients with *DYT*-1 dystonia showed abrupt decrease in movement and disability scores over time. Patients with PKAN revealed relatively higher movement and disability scores preoperatively. The mean scores of the patients with tardive dystonia remained staionary. (PKAN: pantothenate kinase-associated neurodegeneration)

**Fig 3:**
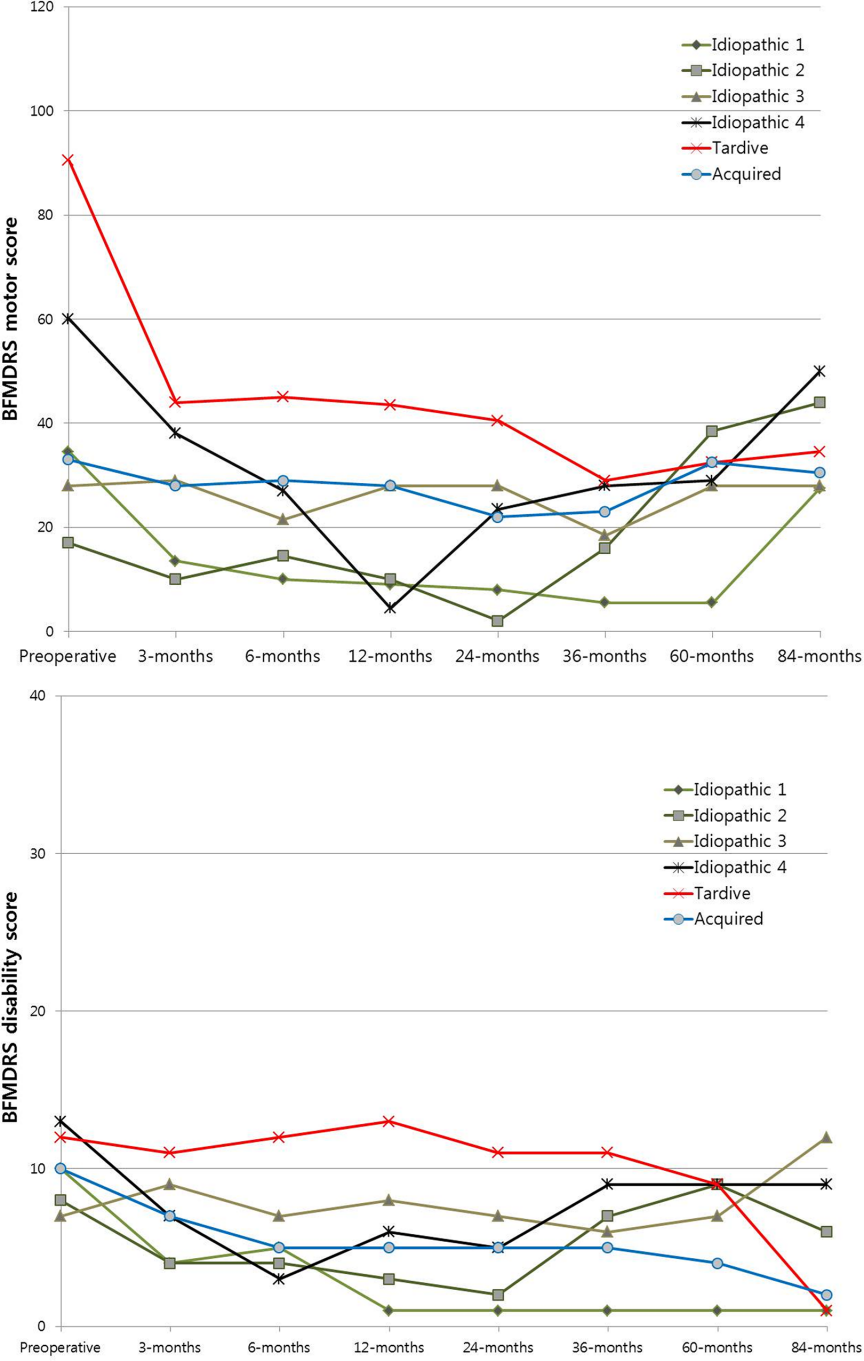
Overall BFMDRS motor and disability scales in 6 patients who underwent 84 months follow up. Of the 6 patients who underwent 84 months follow up, one patient with tardive dystonia showed steady improvement overtime and a patient with acquired dystonia revealed constant scores over time. Except for them, remaining 4 patients with isolated dystonia without known genetic cause showed secondary worsening.

Five patients with PKAN were included in this study. PKAN is an iron metabolism dysregulation caused by a *PANK-*2 mutation, and the patients show a characteristic ‘eye-of-the-tiger sign’ (hypointensity with central hyperintensity in the globus pallidus on T2 images) on brain MRI[30, 31]. Some authors have reported a dramatic improvement in a short time period in PKAN patients [32, 33], But there are debates over the prognosis of the disease. Lim et al. and Krause et al. reported on dystonia patients with PKAN who showed a variable response and did not respond to stimulation [26, 34]. In this study, as shown in Figs 1, 2 and 3 of the 5 PKAN patients showed a substantial decrease in motor and disability scores despite the severely progressed disease state preoperatively.

The patients with acquired dystonia are known to show less favorable response to GPi DBS [23, 35–37]. Speelman et al. reported that GPi DBS was useful in some secondary dystonia patients, but they included many patients with tardive dystonia or PKAN into the secondary dystonia group [28]. In our experience, acquired dystonia patients showed marked improvement during the first 6 months, but the BFMDRS motor and disability scores tended to increase again after this time period.

The criteria for tardive dystonia were proposed by Burke et al. in 1982 [38]. They defined the key feature of tardive dyskinesia as the presence of chronic dystonia, a history of anti-psychotic drug use preceding or concurrent with the onset of dystonia, the absence of a family history of dystonia, and exclusion of other causes of secondary dystonia. Many authors reported a significant benefit of GPi DBS in tardive dystonia patients [21, 24, 37, 39–44] However, others researchers could not find any significant improvement after GPi stimulation in tardive dystonia patients [9, 45]. In accordance with the latter result, the 4 patients diagnosed with tardive dystonia in the present study showed no statistically significant improvement in motor and disability scores. Among them, 2 patients showed marked motor improvement with rates more than 50%, and 1 patient showed a constant disease state during the follow-up period. Only 1 patient suffered disease progression from the focal to generalized form.

### Outcome predictive factors for GPi DBS

We attempted to identify factors for favorable outcomes after GPi DBS. Many authors have reported younger age at the time of surgery and shorter duration of symptoms as predictive factors for a favorable outcome of GPi DBS for dystonia[10, 28, 35, 37, 46, 47]. However, the influence of disease duration is still debated[46]. Less effect of GPi DBS in adults or patients with longer disease duration was explained by chronic deterioration of health over time and skeletal deformity such as the contracture in scoliosis[10, 28]. However, there was no statistical significance of these factors as outcome predictors after GPi DBS in this study.

### Secondary worsening

This report shows a substantial decrease in the mean movement scores until 60 months, while they tended to increase again slightly at 84 months. For cautious interpretation, the overall BFMDRS motor and disability scores for 6 patients who received an 84-month follow up are described in Fig 3. These patients could not regain the benefit, even after extensive reprogramming. Among them, one patient with tardive dystonia showed steady improvement over time, and one patient with acquired dystonia showed constant scores during the follow-up period. Excluding these 2 patients, the remaining 4 patients with isolated dystonia without a known genetic cause experienced aggravation. There are a few studies that have reported this phenomenon as secondary worsening after GPi DBS for dystonia [10, 26, 47][10,26,47], worsening of symptoms following improvement 2–3 years after DBS implantation without an effect of reprogramming [24, 45]. The reason for this secondary worsening has not been identified. One approach would be to reimage these patients to ensure that there was no lead migration.

### Sustained improvement of dystonia after discontinuation of DBS

Five patients showed a sustained benefit following long-term GPi stimulation and subsequent termination of stimulation. Two patients with isolated dystonia without a known genetic cause and one patient each with DYT-1 dystonia, PKAN, and acquired dystonia due to cerebral palsy were included. One patient discontinued the stimulation on his own, and the other patients discontinued the stimulation based on their doctors’ decision because of a consistently improved state for a long time. In all cases with scheduled DBS cessation, the pseudo-turn off test was performed to determine the psychogenic effect before DBS cessation. The median interval between DBS implantation and cessation was 76 months (range, 64–95), and the median follow-up period after DBS cessation was 18 months (range, 7–43). The follow-up data of these patients after DBS cessation were not included in this study.

A few reports have described sustained relief of dystonic symptoms following cessation of DBS of GPi and thalamus [39, 48]. Several hypotheses have been proposed to explain this phenomenon. Starvrinou et al. reported that the patients with secondary segmental dystonia showed improvement in dystonic muscle movement including painful muscle spasms even after DBS termination, and they explained that this phenomenon occurred due to cortical or subcortical neuroplastic changes induced by DBS [49]. In order to explain sustained relief in cervical dystonia and blepharospasm patients for more than one year after cessation of DBS, Vidailhet et al. suggested that DBS therapy might have the capacity to induce a plastic change, which lessens or obviates the need for further treatment in susceptible patients [50]. In 2007, Tisch et al. proposed a theory whereby clinical effects of DBS in dystonia patients had a biphasic response and dystonic symptoms, those with a phasic component improved rapidly, while tonic or fixed components showed delayed improvement [51]. They explained that symptom improvement after DBS termination was caused by delayed improvement.

On the contrary, some authors have reported poor prognosis after DBS termination. Yianni et al. reported a case of a patient who experienced an acute and severe relapse, the so-called ‘rebound effect’, after sudden cessation of DBS [52]. Trottenberg et al. also experienced a similar phenomenon after sudden cessation of stimulation. They speculated that dystonic symptoms recurred because aberrant pathways still persisted and rapidly returned to generate spontaneous low frequency oscillations after DBS was turned off [8]. Coubes et al. also experienced a few cases with a temporarily sustainable and favorable condition after discontinuation of stimulation, but the symptoms recurred within 1 week and disappeared quickly on reactivation of stimulation in all cases [10].

### Adverse events

Previous authors have reported infection, lead revision, and wound problems like granulomas as the common complications after DBS implantation [37]. We experienced 2 cases of infection at the IPG implantation site and one case of intracranial hemorrhage (ICH) in the basal ganglia. In the 2 patients with infection, the IPG device had to be removed. In one patient, the IPG device was reinserted and the patient’s dystonic symptoms improved. The other patient suffered sustained infection at the IPG implantation site and underwent a bilateral gamma-knife pallidotomy (60 Gy in 3 fractionations). He is showing markedly improved movement and daily activity.

We stopped the procedure when ICH of the basal ganglia occurred in a patient during lead insertion. The patient developed new neurologic deficits such as hemiparesis and facial palsy, but the symptoms resolved after conservative management and physical rehabilitation. After 1 year, DBS implantation was performed and his dystonic symptoms almost disappeared. This patient was included in this study after the second DBS implantation surgery.

### Limitations of this study

The main limitations of this study are that it has a retrospective design along with a small sample size and the follow-up duration is variable among patients. Also, the BFMDRS scores were estimated in an unblinded manner. The difference in improvement rates between subgroups could be partially related to the distribution of the follow-up duration, because most of the data were clustered during the period within 24 months after DBS implantation. A second limitation is the problem of reflection of the scoring system. We used only BFMDRS motor and disability scores for all subtypes of patients; hence, the results would be different from those in other studies that applied specific scales to each disease entity. Also, there is a risk of type II error in our conclusion on the predictive factors for dystonia. A third limitation is that eight patients also had a severe depressive mood disorder at the time of DBS implantation. Some studies have reported ongoing depression and anxiety after GPi DBS, despite significant dystonia improvement [10, 29, 50]. The movement and disability scores would be underestimated due to noncooperation caused by depression and anxiety. Unlike in the cases of with Parkinson disease, formal evaluation of mood was not performed in the patients with dystonia.

Further double-blind, prospective studies and careful analysis of stimulation targets and postoperative results seem to be mandatory for better selection of patients for GPi DBS.

## Conclusion

In conclusion, this study demonstrated that GPi DBS is a safe and effective therapeutic method for treatment of both movement and disability in dystonia patients. A favorable outcome is expected in patients with DYT-1 dystonia and isolated dystonia without a known genetic cause. However, this study has some limitations such as the retrospective design along with a small sample size and that it was performed at a single-center.

## Supporting Information

S1 Table The median DBS stimulation parameters of specific subgroups. (DOCX)

S2 Table The data set which was used in this study. (XLSX)

## References

[1] Fahn S. Concept and classification of dystonia. Adv Neurol. 1988;50:1. 3041755

[2] Müller J, Kiechl S, Wenning G, Seppi K, Willeit J, Gasperi A, et al The prevalence of primary dystonia in the general community. Neurology. 2002;59(6):941–3. 12297587 10.1212/01.wnl.0000026474.12594.0d

[3] Albanese A, Bhatia K, Bressman SB, DeLong MR, Fahn S, Fung VS, et al Phenomenology and classification of dystonia: a consensus update. Mov Disord. 2013;28(7):863–73. 10.1002/mds.25475 23649720 PMC3729880

[4] Müller J, Kemmler G, Wissel J, Schneider A, Voller B, Grossmann J, et al The impact of blepharospasm and cervical dystonia on health-related quality of life and depression. Journal of neurology. 2002;249(7):842–6. 12140667 10.1007/s00415-002-0733-1

[5] Lozano AM, Kumar R, Gross R, Giladi N, Hutchison W, Dostrovsky J, et al Globus pallidus internus pallidotomy for generalized dystonia. Movement disorders. 1997;12(6):865–70. 9399208 10.1002/mds.870120606

[6] Vitek JL, Bakay RA, Hashimoto T, Kaneoke Y, Mewes K, Zhang JY, et al Microelectrode-guided pallidotomy: technical approach and its application in medically intractable Parkinson's disease. Journal of neurosurgery. 1998;88(6):1027–43. 9609298 10.3171/jns.1998.88.6.1027

[7] Toda H, Hamani C, Lozano A. Deep brain stimulation in the treatment of dyskinesia and dystonia. Neurosurg Focus. 2004;17(1):9–13. 10.3171/foc.2004.17.1.2 15264771

[8] Trottenberg T, Meissner W, Arnold G, Einhaupl KM, Kupsch A, Kabus C, et al Neurostimulation of the ventral intermediate thalamic nucleus in inherited myoclonus‐dystonia Syndrome. Movement disorders. 2001;16(4):769–71. 11481711 10.1002/mds.1119

[9] Krauss JK, Loher TJ, Weigel R, Capelle HH, Weber S, Burgunder J-M. Chronic stimulation of the globus pallidus internus for treatment of non-dYT1 generalized dystonia and choreoathetosis: 2-year follow up. J Neurosurg. 2003;98(4):785–92. 12691403 10.3171/jns.2003.98.4.0785

[10] Coubes P, Cif L, El Fertit H, Hemm S, Vayssiere N, Serrat S, et al Electrical stimulation of the globus pallidus internus in patients with primary generalized dystonia: long-term results. J Neurosurg. 2004;101(2):189–94. 10.3171/jns.2004.101.2.0189 15309907

[11] Vidailhet M, Vercueil L, Houeto J-L, Krystkowiak P, Benabid A-L, Cornu P, et al Bilateral deep-brain stimulation of the globus pallidus in primary generalized dystonia. N Engl J Med. 2005;352(5):459–67. 15689584 10.1056/NEJMoa042187

[12] Coubes P, Roubertie A, Vayssiere N, Hemm S, Echenne B. Treatment of DYT1-generalised dystonia by stimulation of the internal globus pallidus. The Lancet. 2000;355(9222):2220–1. 10.1016/S0140-6736(00)02410-7 10881900

[13] Kupsch A, Benecke R, Müller J, Trottenberg T, Schneider G-H, Poewe W, et al Pallidal deep-brain stimulation in primary generalized or segmental dystonia. N Engl J Med. 2006;355(19):1978–90. 17093249 10.1056/NEJMoa063618

[14] Cif L, El Fertit H, Vayssiere N, Hemm S, Hardouin E, Gannau A, et al Treatment of dystonic syndromes by chronic electrical stimulation of the internal globus pallidus. Journal of neurosurgical sciences. 2003;47(1):52–5. 12900733

[15] Volkmann J, Wolters A, Kupsch A, Müller J, Kühn AA, Schneider G-H, et al Pallidal deep brain stimulation in patients with primary generalised or segmental dystonia: 5-year follow-up of a randomised trial. The Lancet Neurology. 2012;11(12):1029–38. 10.1016/S1474-4422(12)70257-0 23123071

[16] Brüggemann N, Kühn A, Schneider SA, Kamm C, Wolters A, Krause P, et al Short-and long-term outcome of chronic pallidal neurostimulation in monogenic isolated dystonia. Neurology. 2015;84(9):895–903. 10.1212/WNL.0000000000001312 25653290 PMC6170184

[17] Burke RE, Fahn S, Marsden CD, Bressman SB, Moskowitz C, Friedman J. Validity and reliability of a rating scale for the primary torsion dystonias. Neurology. 1985;35(1):73–. 3966004 10.1212/wnl.35.1.73

[18] Krauss J. Deep brain stimulation for cervical dystonia. Journal of Neurology, Neurosurgery & Psychiatry. 2003;74(11):1598–. 10.1136/jnnp.74.11.1598 PMC1738222 14617734

[19] Coubes P, Vayssiere N, El Fertit H, Hemm S, Cif L, Kienlen J, et al Deep brain stimulation for dystonia. Surgical technique. Stereotactic and functional neurosurgery. 2001;78(3–4):183–91. 10.1159/000068962 12652042

[20] Bereznai B, Steude U, Seelos K, Bötzel K. Chronic high‐frequency globus pallidus internus stimulation in different types of dystonia: A clinical, video, and MRI report of six patients presenting with segmental, cervical, and generalized dystonia. Movement disorders. 2002;17(1):138–44. 11835451 10.1002/mds.1250

[21] Trottenberg T, Paul G, Meissner W, Maier-Hauff K, Taschner C, Kupsch A. Pallidal and thalamic neurostimulation in severe tardive dystonia. Journal of Neurology, Neurosurgery & Psychiatry. 2001;70(4):557–9. 10.1136/jnnp.70.4.557 PMC1737288 11254790

[22] Alterman R, Miravite J, Weisz D, Shils J, Bressman S, Tagliati M. Sixty hertz pallidal deep brain stimulation for primary torsion dystonia. Neurology. 2007;69(7):681–8. 17698790 10.1212/01.wnl.0000267430.95106.ff

[23] Tronnier VM, Fogel W. Pallidal stimulation for generalized dystonia: report of three cases. Journal of neurosurgery. 2000;92(3):453–6. 10701533 10.3171/jns.2000.92.3.0453

[24] Kosel M, Sturm V, Frick C, Lenartz D, Zeidler G, Brodesser D, et al Mood improvement after deep brain stimulation of the internal globus pallidus for tardive dyskinesia in a patient suffering from major depression. Journal of psychiatric research. 2007;41(9):801–3. 16962613 10.1016/j.jpsychires.2006.07.010

[25] Tisch S, Zrinzo L, Limousin P, Bhatia KP, Quinn N, Ashkan K, et al Effect of electrode contact location on clinical efficacy of pallidal deep brain stimulation in primary generalised dystonia. Journal of Neurology, Neurosurgery & Psychiatry. 2007;78(12):1314–9. 10.1136/jnnp.2006.109694 PMC2095629 17442760

[26] Krause M, Fogel W, Kloss M, Rasche D, Volkmann J, Tronnier V. Pallidal stimulation for dystonia. Neurosurgery. 2004;55(6):1361–70. 15574217 10.1227/01.neu.0000143331.86101.5e

[27] Ostrem JL, Starr PA. Treatment of dystonia with deep brain stimulation. Neurotherapeutics. 2008;5(2):320–30. 10.1016/j.nurt.2008.01.002 18394573 PMC5084173

[28] Speelman J, Contarino M, Schuurman P, Tijssen M, De Bie R. Deep brain stimulation for dystonia: patient selection and outcomes. European Journal of Neurology. 2010;17(s1):102–6. 20590816 10.1111/j.1468-1331.2010.03060.x

[29] Jahanshahi M, Torkamani M, Beigi M, Wilkinson L, Page D, Madeley L, et al Pallidal stimulation for primary generalised dystonia: effect on cognition, mood and quality of life. Journal of neurology. 2014;261(1):164–73. 10.1007/s00415-013-7161-2 24178706 PMC3895192

[30] Hayflick SJ, Westaway SK, Levinson B, Zhou B, Johnson MA, Ching KH, et al Genetic, clinical, and radiographic delineation of Hallervorden–Spatz syndrome. New England Journal of Medicine. 2003;348(1):33–40. 12510040 10.1056/NEJMoa020817

[31] Zhou B, Westaway SK, Levinson B, Johnson MA, Gitschier J, Hayflick SJ. A novel pantothenate kinase gene (PANK2) is defective in Hallervorden-Spatz syndrome. Nature genetics. 2001;28(4):345–9. 11479594 10.1038/ng572

[32] Justesen CR, Penn RD, Kroin JS, Egel RT. Stereotactic pallidotomy in a child with Hallervorden-Spatz disease: Case report. Journal of neurosurgery. 1999;90(3):551–4. 10067928 10.3171/jns.1999.90.3.0551

[33] Vercueil L, Pollak P, Fraix V, Caputo E, Moro E, Benazzouz A, et al Deep brain stimulation in the treatment of severe dystonia. Journal of neurology. 2001;248(8):695–700. 11569899 10.1007/s004150170116

[34] Lim B, Ki CS, Cho A, Hwang H, Kim K, Hwang Y, et al Pantothenate kinase‐associated neurodegeneration in Korea: recurrent R440P mutation in PANK2 and outcome of deep brain stimulation. European Journal of Neurology. 2012;19(4):556–61. 10.1111/j.1468-1331.2011.03589.x 22103354

[35] Vidailhet M, Jutras M-F, Grabli D, Roze E. Deep brain stimulation for dystonia. J Neurol Neurosurg Psychiatry. 2012:jnnp-2011-301714. 10.1136/jnnp-2011-301714 23154125

[36] Lee JY, Deogaonkar M, Rezai A. Deep brain stimulation of globus pallidus internus for dystonia. Parkinsonism Relat Disord. 2007;13(5):261–5. 17081796 10.1016/j.parkreldis.2006.07.020

[37] FitzGerald J, Rosendal F, De Pennington N, Joint C, Forrow B, Fletcher C, et al Long-term outcome of deep brain stimulation in generalised dystonia: a series of 60 cases. J Neurol Neurosurg Psychiatry. 2014:jnnp-2013-306833. 10.1136/jnnp-2013-306833 24691580

[38] Burke RE, Fahn S, Jankovic J, Marsden C, Lang AE, Gollomp S, et al Tardive dystonia Late‐onset and persistent dystonia caused by antipsychotic drugs. Neurology. 1982;32(12):1335–. 6128697 10.1212/wnl.32.12.1335

[39] Johnsen M, Wester K. Full remission of tardive dyskinesia following general anaesthesia. Journal of neurology. 2002;249(5):622–5. 12021954 10.1007/s004150200073

[40] Kefalopoulou Z, Paschali A, Markaki E, Vassilakos P, Ellul J, Constantoyannis C. A double‐blind study on a patient with tardive dyskinesia treated with pallidal deep brain stimulation. Acta neurologica Scandinavica. 2009;119(4):269–73. 10.1111/j.1600-0404.2008.01115.x 18976318

[41] Eltahawy HA, Feinstein A, Khan F, Saint‐Cyr J, Lang AE, Lozano AM. Bilateral globus pallidus internus deep brain stimulation in tardive dyskinesia: a case report. Movement disorders. 2004;19(8):969–72. 15300668 10.1002/mds.20092

[42] Schrader C, Peschel T, Petermeyer M, Dengler R, Hellwig D. Unilateral deep brain stimulation of the internal globus pallidus alleviates tardive dyskinesia. Movement disorders. 2004;19(5):583–5. 15133825 10.1002/mds.10705

[43] Damier P, Thobois S, Witjas T, Cuny E, Derost P, Raoul S, et al Bilateral deep brain stimulation of the globus pallidus to treat tardive dyskinesia. Archives of general psychiatry. 2007;64(2):170–6. 17283284 10.1001/archpsyc.64.2.170

[44] Weetman J, Anderson I, Gregory R, Gill S. Bilateral posteroventral pallidotomy for severe antipsychotic induced tardive dyskinesia and dystonia. Journal of Neurology, Neurosurgery & Psychiatry. 1997;63(4):554–6. 10.1136/jnnp.63.4.554a PMC2169756 9343153

[45] Starr PA, Turner RS, Rau G, Lindsey N, Heath S, Volz M, et al Microelectrode-guided implantation of deep brain stimulators into the globus pallidus internus for dystonia: techniques, electrode locations, and outcomes. Journal of neurosurgery. 2006;104(4):488–501. 16619651 10.3171/jns.2006.104.4.488

[46] Valldeoriola F, Regidor I, Mínguez-Castellanos A, Lezcano E, García-Ruiz P, Rojo A, et al Efficacy and safety of pallidal stimulation in primary dystonia: results of the Spanish multicentric study. Journal of Neurology, Neurosurgery & Psychiatry. 2010;81(1):65–9. 10.1136/jnnp.2009.174342 19744963

[47] Loher T, Capelle H-H, Kaelin-Lang A, Weber S, Weigel R, Burgunder J, et al Deep brain stimulation for dystonia: outcome at long-term follow-up. Journal of neurology. 2008;255(6):881–4. 10.1007/s00415-008-0798-6 18338193

[48] Foote KD, Sanchez JC, Okun MS. Staged deep brain stimulation for refractory craniofacial dystonia with blepharospasm: case report and physiology. Neurosurgery. 2005;56(2):E415 15670394 10.1227/01.neu.0000147978.67424.42

[49] Stavrinou LC, Boviatsis EJ, Stathis P, Leonardos A, Panourias IG, Sakas DE. Sustained relief after discontinuation of DBS for dystonia: implications for the possible role of synaptic plasticity and cortical reorganization. Journal of neurological surgery Part A, Central European neurosurgery. 2012;73(3):175–8; discussion 8–9. Epub 2011/06/02. 10.1055/s-0032-1313590 . 21630190

[50] Vidailhet M, Vercueil L, Houeto J-L, Krystkowiak P, Lagrange C, Yelnik J, et al Bilateral, pallidal, deep-brain stimulation in primary generalised dystonia: a prospective 3 year follow-up study. The Lancet Neurology. 2007;6(3):223–9. 17303528 10.1016/S1474-4422(07)70035-2

[51] Tisch S, Rothwell JC, Limousin P, Hariz MI, Corcos DM. The physiological effects of pallidal deep brain stimulation in dystonia. Neural Systems and Rehabilitation Engineering, IEEE Transactions on. 2007;15(2):166–72. 10.1109/TNSRE.2007.896994 17601185

[52] Yianni J, Bain P, Giladi N, Auca M, Gregory R, Joint C, et al Globus pallidus internus deep brain stimulation for dystonic conditions: a prospective audit. Movement disorders. 2003;18(4):436–42. 12671953 10.1002/mds.10380

