# Supplementary material for: Long-Term Clinical Outcome of Internal Globus Pallidus Deep Brain Stimulation for Dystonia
Source: PLoS One. 2016 Jan 8;11(1):e0146644. doi: 10.1371/journal.pone.0146644 (PMC4706355; doi:10.1371/journal.pone.0146644)
Supplement: S1 Table — (DOCX) [file pone.0146644.s001.docx]

|  | DYT | PKAN | Secondary | Isolated without known genetic cause |
| --- | --- | --- | --- | --- |
| Initial parameter (median) |  |  |  |  |
| Lt |  |  |  |  |
| pulse width (㎲) | 60 | 60 | 60 | 60 |
| Frequency (Hz) | 130 | 130 | 130 | 130 |
| Amplitude (V) | 3.15 | 1.0 | 2.7 | 2.15 |
| Rt |  |  |  |  |
| pulse width (㎲) | 60 | 60 | 60 | 60 |
| Frequency (Hz) | 130 | 130 | 130 | 130 |
| Amplitude (V) | 3.4 | 1.0 | 2.8 | 2.20 |
| Final parameter (median) |  |  |  |  |
| Lt |  |  |  |  |
| pulse width (㎲) | 60 | 120 | 60 | 105 |
| Frequency (Hz) | 130 | 130 | 130 | 130 |
| Amplitude (V) | 3.5 | 3.5 | 3.05 | 3.0 |
| Rt |  |  |  |  |
| pulse width (㎲) | 60 | 90 | 60 | 90 |
| Frequency (Hz) | 130 | 130 | 130 | 130 |
| Amplitude (V) | 3.0 | 2.5 | 2.8 | 2.7 |
